# Spontaneous Splenic Rupture as a Rare Initial Presentation in an Acute Lymphoblastic Leukemia Patient

**DOI:** 10.3390/diagnostics9040152

**Published:** 2019-10-18

**Authors:** Meng-Yu Wu, Woei-Yau Kao, Cheng-Yi Chan, Giou-Teng Yiang, Wan-Ting Liao, Chien-Sheng Chen

**Affiliations:** 1 Department of Emergency Medicine, Taipei Tzu Chi Hospital, Buddhist Tzu Chi Medical Foundation, New Taipei 231, Taiwan; skyshangrila@gmail.com (M.-Y.W.); gtyiang@gmail.com (G.-T.Y.); 2 Department of Emergency Medicine, School of Medicine, Tzu Chi University, Hualien 970, Taiwan; 3 Division of Hematology and Oncology, Department of Medicine, Taipei Tzu Chi Hospital, Buddhist Tzu Chi Medical Foundation, Taipei 231, Taiwan; wykao@seed.net.tw; 4 Department of Medicine, School of Medicine, Tzu Chi University, Hualien 970, Taiwan; 5 Department of Medical Imaging, Taipei Tzu Chi Hospital, the Buddhist Tzu Chi Medical Foundation, New Taipei 231 Taiwan; ccy983998@gmail.com; 6 Department of Medical Imaging, School of Medicine, Tzu Chi University, Hualien 970, Taiwan; 7 Institute of Medicine, Chung Shan Medical University, Taichung 402, Taiwan; 8 Chinese Medicine Department, Show Chwan Memorial Hospital, Changhua 500, Taiwan

**Keywords:** splenic rupture, acute lymphoblastic leukemia, acute abdomen, computed tomography

## Abstract

A spontaneous rupture of the spleen is a rare but critical diagnosis of an acute abdomen, which may accompany unspecific symptoms mimicking acute pancreatitis, rupture of aortic aneurism, or acute coronary syndrome, delaying diagnosis and treatment. In patients that have experienced a severe spleen rupture, hypovolemic shock may cause catastrophic clinical outcomes. Therefore, early diagnosis is very important in order for physicians to declare the etiology for prevention and timely correction of the shock status. Several causes of spontaneous splenic rupture have been reported, including infection, vasculitis, pancreatitis, or hematological malignancies. Acute lymphoblastic leukemia (ALL) remains a rare but important cause of non-traumatic splenic rupture that physicians are required to assess for. Here, we describe a case presenting an acute abdomen due to spontaneous spleen rupture as the first manifestation. The purpose of this case report was to highlight the importance of considering spontaneous ruptures of the spleen as a rare but critical differential diagnosis of an acute abdomen, especially in patients with acute lymphoblastic leukemia.

## 1. Introduction

Spontaneous splenic ruptures are a rare clinical phenomenon that may lead to hemodynamic instability due to hypovolemic shock, and may also be difficult to diagnose as a first manifestation in an emergency department [1]. The incidences of “true” spontaneous rupture of spleen have been low in previous reports. In current concepts, non-traumatic splenic ruptures are usually pathological ruptures associated with diseases. Many etiologies of non-traumatic splenic ruptures have been reported, such as infections, vasculitis, pancreatitis, or hematological malignancies [2,3,4]. Acute lymphoblastic leukemia (ALL) remains a rare cause of non-traumatic splenic rupture that physicians are required to assess for. Due to the unspecific symptoms, a spontaneous splenic rupture may mimic perforated viscus, acute pancreatitis, rupture of aortic aneurysm, or acute coronary syndrome, delaying the diagnosis. A detailed pathophysiology of spontaneous splenic ruptures is still unclear. In a few previous reports, it has been noted that spontaneous splenic rupture may be induced by an expanding subcapsular hematoma that eventually tears the capsule through a Valsalva maneuver such as a cough, sneeze, or vomiting, which, in turn, increases the venous portal pressure or compression of the spleen [5,6,7]. Therefore, early diagnosis is very important in order for emergency physicians to stabilize hypovolemic shock and declare the etiology of a spontaneous splenic rupture. We describe a unique case of an acute abdomen due to a spontaneous splenic rupture as a first manifestation in an ALL patient. The etiology and clinical features of spontaneous splenic rupture are discussed.

## 2. Case Presentation

A 23-year-old male was transferred to our emergency department from a different hospital presenting sudden onset progressive abdominal pain. He was suffering from intermittent fever and progressive abdominal pain for 1 day. He confirmed that he had not undergone any traumatic injury recently. There were no other accompanying symptoms, such as nausea, vomiting, diarrhea, tarry stool, or bloody stool. His pain was localized in the epigastric area without radiation to the back or left shoulder. On admission, his temperature was 36.7 °C, blood pressure was 102/86 mmHg, and heart rate was 98 beats/min. A physical examination revealed a soft abdomen without any ecchymosis, Cullen’s, or Grey Turner’s signs. There was no local tenderness, muscle guarding, Murphy’s sign, or McBurney’s point tenderness. The laboratory test revealed severe leukocytosis, thrombocytopenia, and elevated lactic dehydrogenase (LDH). The detailed laboratory results are listed in Table 1. Emergency hydroxyurea 1000 mg was administered for hematological disorders.

**Figure 1:**
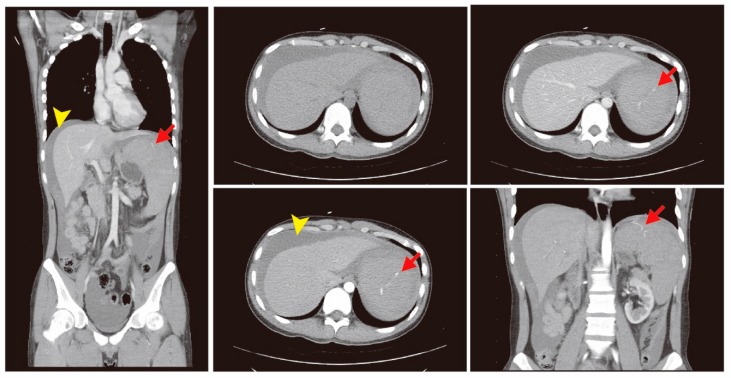
The abdominal contrast enhanced computed tomography (CT) showed splenomegaly and a splenic rupture (red arrow) with hemoperitoneum (yellow arrow head).

**Figure 2:**
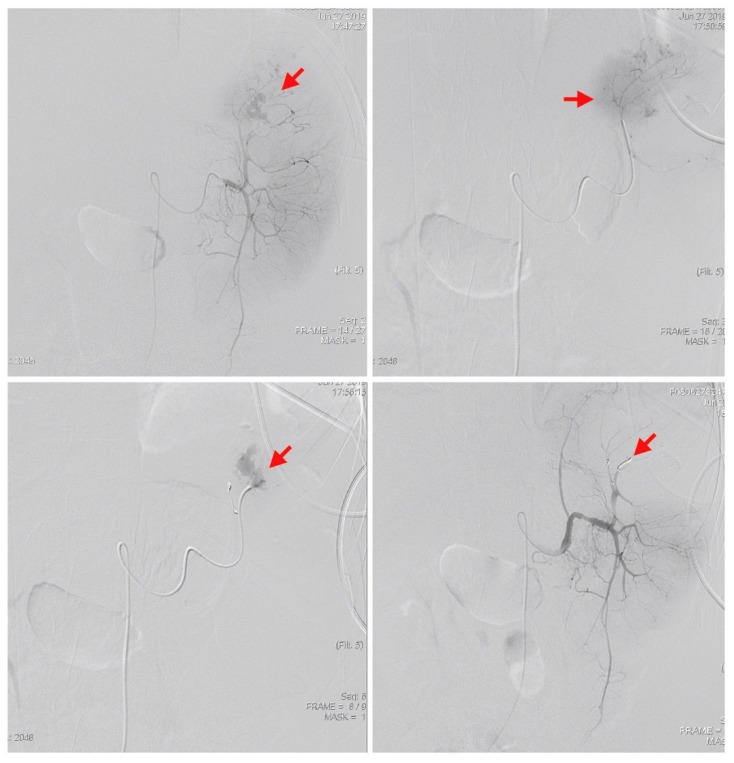
An emergency transcatheter arterial embolization was performed using two coils to control splenic hemorrhage (red arrows).

The bedside ultrasound revealed much ascites, which confirmed the emergency abdominal contrast enhanced computed tomography (CT) in the previous transfer hospital and showed splenomegaly and splenic rupture with hemoperitoneum (Figure 1). Adequate fluid resuscitation and emergency blood transfusion were performed to correct the hypovolemic shock status. Transamine (1000 mg) and vitamin K1 (10 mg) were administered to control the bleeding. The opioid agent morphine (10 mg) was used to control progressive abdominal pain. An emergency transcatheter arterial embolization was performed to control persistent splenic hemorrhage (Figure 2).

**Figure 3:**
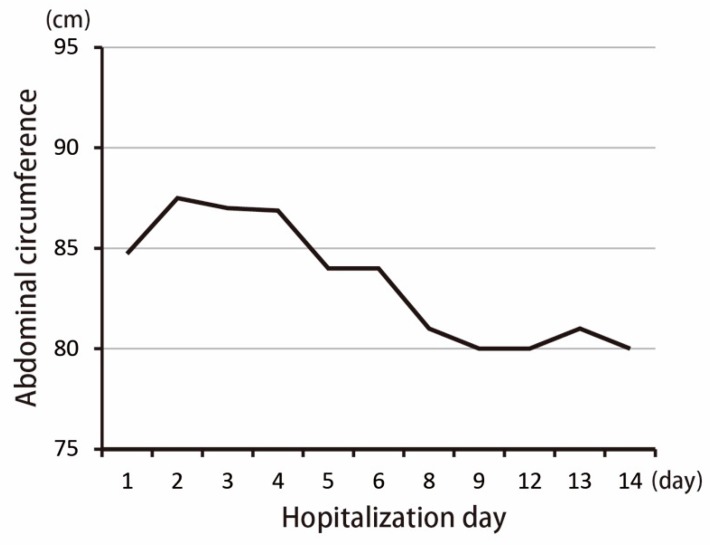
Change in abdominal circumference after hospitalization.

**Figure 4:**
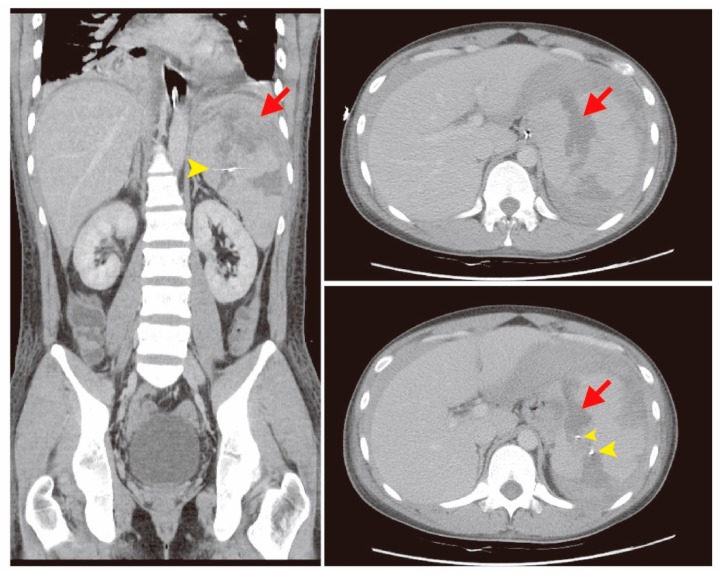
In the follow-up computed tomography (CT) scan, partial infarct of the spleen (red arrows) with two-coil embolization was noted, with improved hemoperitoneum (yellow arrow heads).

The patient was hospitalized in the intensive care unit (ICU) for post-embolization care. There was no progressive abdominal pain. The follow-up abdominal circumference was decreased, which reflected that intra-abdominal bleeding was under control due to a decreased amount of ascites (Figure 3). The follow-up CT scan revealed the spleen hemorrhage had been controlled through coil embolization (Figure 4). The hemoperitoneum also improved. A bone marrow examination showed sheets of small blasts with scant cytoplasm. Monotonous blasts comprised more than 95% of nucleated cells. ALL with scant cytoplasm was diagnosed with BCR-ABL (+), Periodic acid-Schiff (PAS) (−), peroxidase (−), TdT (< 1%), CD34 (< 1%), CD117 (< 1%), MPO (+, 60%), and CD20 (+, 5–10%). Broad-spectrum antibiotics, including Meropenem, Mycamine, and Teicoplanin, were administered to treat a severe immunocompromised infection due to persistent fever. After supportive care, extubation was performed and the patient stabilized. Target therapy with the agent Dasatinib (50 mg three times per day) combined with chemotherapy agents, including Vincristine and Methotrexate, controlled the progression of ALL. Intrathecal chemotherapy with Methotrexate (15 mg) and methylprednisolone (40 mg) was administered. Bone marrow aspiration and cytology were performed again and revealed normal cellularity. There were no significant side effects or complications. He was regularly followed-up at the outpatient clinic. The follow-up laboratory data is shown in Table 2. A patient consent form has been obtained from the patient.

## 3. Discussion

Spontaneous splenic rupture is a rare but devastating etiology of an acute abdomen in ALL patients. In a review by P. Renzulli et al. [8], the authors summarized 845 atraumatic splenic rupture patients from previous reports dating from 1980 to 2008. Atraumatic, pathological ruptures were found in 93% of the cases. In etiological analysis, the neoplastic cause accounted for 30.3% of cases, followed by infection in 27.3% of cases, inflammation in 20.0% of cases, and drug/treatment-related causes in 9.2% of cases. In the analyses of malignant hematological disorders, non-Hodgkin’s lymphoma was the condition most commonly associated with splenic ruptures (55%), followed by myeloproliferative disorders (24%) and ALL (12%). Several risk factors of pathologic splenic ruptures in patients with hematologic malignancies have been identified, including age, gender, severe splenomegaly, disease type, and previous or present use of G-CSF [1,9,10]. In treatment analysis, 84.1% patients received total splenectomies for spontaneous splenic ruptures, followed by 1.2% in organ-preserving surgery and 14.7% in conservative measures. Three factors involved in the mortality rate of atraumatic-pathological ruptures included splenomegaly, age above 40 years, and neoplastic disorders. In our patient, the hemodynamic condition was stable after resuscitation. The less invasive treatment, emergency transcatheter arterial embolization, was first performed to prevent post-splenectomy infections due to loss of immunological function in the spleen. Based on the World Society of Emergency Surgery (WSES) classification of splenic injury, our patient was classified into the moderate type. Angiography was also initially suggested. However, there was no strong evidence of a therapeutic strategy for hematological disorder- induced spontaneous splenic rupture, as it was an uncommon case.

The detailed mechanism of pathologic splenic rupture in hematological disorders remains unclear. Two major theories have been reported to explain the pathophysiology of spontaneous splenic ruptures in acute lymphoblastic leukemia. One theory demonstrates that the splenic structure is destroyed by the severe infiltration of malignant cells. The direct infiltration leads to overloading the organ beyond its capability of compensation and expansion, which may potentially promote a rupture of the splenic capsule. Infiltration of malignant cells also causes progressive splenomegaly with a potential risk of splenic rupture due to accidental trauma. Another theory promotes that the rapid expansion of the organ causes insufficient blood supply, which may lead to local infarction, and the infarction site may become a weak point [11,12,13]. In addition, hyperleukocytosis in patients with hematological disorders may also trigger severe disseminated intravascular coagulation (DIC) and leukostasis, which are the two main risk factors that promote hemorrhaging [14]. These theories explain the mechanism of spontaneous splenic ruptures in ALL. In ALL patients, hyperleukocytosis may lead to rapid splenomegaly and distortion of the splenic architecture, easily causing splenic infarction, hemorrhaging, and capsule rupture [15].

The diagnosis of splenic rupture must be considered in all ALL patients who presented with sudden onset left upper abdominal pain, a persistent hemodynamic unstable condition, or acute anemia [16]. However, early diagnosis with an adequate therapeutic strategy for pathologic splenic rupture is difficult due to nonspecific symptoms. Images play an important role in addressing the acute abdomen. Free intraperitoneal fluid is an important hint that can suggest splenic rupture. In a systematic review analysis, the final diagnosis of splenic rupture in 42.3% patients was made via laparotomy, followed by 32.4% via CT, 18.6% via ultrasonography, 0.7% via scintigraphy, and 0.3% via angiography; 5.2% were diagnosed during post-mortem examination [8]. In our case, the abdominal CT scan was diagnostic, and the emergency transcatheter arterial embolism was performed immediately to control the hemorrhage. Although the prognosis in splenic rupture is poor, aggressive management with early transcatheter arterial embolism can correct the hypovolemic shock. We presented this rare case of pathologic splenic rupture in an ALL patient to highlight an uncommon but important etiology that should be kept in mind when treating patients with an acute abdomen. Early management can prevent poor clinical prognosis.

## Figures and Tables

**Table 1:** Laboratory evaluation of the patient.

Variables	Normal Range	Patient Data	Variables	Normal Range	Patient Data
White blood cells (WBC)	3.5–11 (×10^9^/L)	102.04	Blood urine nitrogen	7–18 mg/dL	21
Segment form neutrophils	45–70%	6.0	Creatinine	0.55–1.02 mg/dL	1.3
Lymphocytes	25–40%	3.0	Sodium	136–145 mmol/L	140
Eosinophils	1–3%	0.0	Potassium	3.5–5.1 mmol/L	4.3
Monocytes	2–8%	1.0	Calcium	2.12–2.52 mmol/L	1.91
Nuclear red blood cells	0/100WBC	1.0	Glucose	70–100 mg/dL	145
Blast cell	0%	88.0	Alanine aminotransferase	16–63 U/L	51
Hemoglobin	12–16 g/dL	9.4	Lipase	73–393 IU/L	206
Platelet counts	150–400 (×10^9^/L)	32	Creatine kinase	26–192 IU/L	184
Prothrombin time	8.0–12.0 s	12.7	C-reactive protein (CRP)	0–0.33 mg/dL	0.66
Partial thromboplastin time	23.9–35.5 s	30.6			
Lactic dehydrogenase (LDH)	85–227 IU/L	894			

**Table 2:** The follow-up laboratory data of this patient.

Variables	Normal Range	Day 0	Day 1	Day 2	Day 3	Day 4	Day 5	Day 6	Day 7	Day 8	Day 9	Day 11	Day 12
WBC	3.5–11 (× 10^9^/L)	102.04	22.82	5.69	3.9	2.87	2.01	1.92	2.03	2.39	1.64	0.99	1.34
RBC	4.5–5.9 (× 10^6^/uL)	3.43	3.13	2.84	2.86	2.84	3.42	3.37	3.55	3.85	3.8	3.54	3.61
Hb	12–16 g/dL	9.4	8.7	8.1	8.4	8.1	9.7	9.7	10.1	11.3	11	10.2	10.2
PL	150–400 (× 10^9^/L)	32	85	90	53	68	43	29	70	42	42	85	84
N. band	0–3%	0	5	3	6	7	0	5	0	1	0	1	3
N. seg.	40–75%	6	6	16	24	24	52	46	58.1	56	59	52	40
Lym.	20–45%	3	37	55	34	38	35	41	34	37	38	39	50
Mono.	2–10%	1	1	2	3	1	2	1	3	2	2	3	3
Eosin.	1–6%	0	0	3	0	0	0	1	4.9	0	1	3	4
Baso.	0–1%	1	0	0	1	0	0	0	0	0	0	0	0
Blast	0–0%	88	48	18	26	24	9	2	0	1	0	0	0

WBC: white cell count, RBC: red cell count, Hb: hemoglobin, PL: platelet counts, N. band: band-form neutrophils, N. seg: segment-form neutrophils, Lym.: lymphocytes, Mono.: monocytes, Eosin.: eosinophils, Baso.: basophils, Blast: blast cells.
